# Efficacy and biomarker analysis of neoadjuvant disitamab vedotin (RC48‐ADC) combined immunotherapy in patients with muscle‐invasive bladder cancer: A multi‐center real‐world study

**DOI:** 10.1002/imt2.70033

**Published:** 2025-04-14

**Authors:** Jiao Hu, Luzhe Yan, Jinhui Liu, Minfeng Chen, Peihua Liu, Dingshan Deng, Chaobin Zhang, Yunbo He, Benyi Fan, Huihuang Li, Guanghui Gong, Jiatong Xiao, Ruizhe Wang, Xiao Guan, Shiyu Tong, Yangle Li, Nannan Li, Zhiwang Tang, Teng Zhang, Hao Li, Bin Huang, Ning Gao, Wei He, Zhiyong Cai, Yifan Liu, Zefu Liu, Yu Gan, Yu Cui, Yuanqing Dai, Yi Cai, Zhenyu Nie, Zhenyu Ou, Jinbo Chen, Xiongbing Zu

**Affiliations:** ^1^ Department of Urology, Xiangya Hospital Central South University Changsha China; ^2^ National Clinical Research Center for Geriatric Disorders, Xiangya Hospital Central South University Changsha China; ^3^ Furong Laboratory Changsha China; ^4^ Department of Pathology, Xiangya Hospital Central South University Changsha China; ^5^ Department of Urology The First Hospital of Changsha Changsha China; ^6^ Department of Urology First People's Hospital of Guiyang Guiyang China; ^7^ Department of Urology Xiangya Boai Rehabilitation Hospital Changsha China; ^8^ Department of Urology, Hunan Provincial People's Hospital the First Affiliated Hospital of Hunan Normal University Changsha China

## Abstract

In this study, 102 cisplatin‐ineligible patients with muscle‐invasive bladder cancer (MIBC) who received neoadjuvant RC48‐ADC combined with immunotherapy were included. We evaluated the pathological responses and explored multiple clinical characteristics to identify independent predictive indicators of the efficacy. The results showed that neoadjuvant RC48‐ADC combined with immunotherapy had promising efficacy. Furthermore, we collected 11 MIBC samples and performed single‐cell RNA sequencing. All BLCA epithelial cells were identified as four subclusters. We conducted differential gene expression/functional enrichment analysis, cell proportion analysis, cell cycle analysis, CNV analysis, and pseudotemporal analysis on all tumor cells to evaluate potential efficacy‐predictive biomarkers and the evolutionary patterns of tumor cells during neoadjuvant treatment. The results indicated that the combined detection of HER2 and HSPA1A expression in C3 subcluster based on single‐cell RNA sequencing is a potential strategy for predicting efficacy. In addition, C3 plays a dominant role in the emergence of drug‐resistance during the evolution of BLCA epithelial cells.

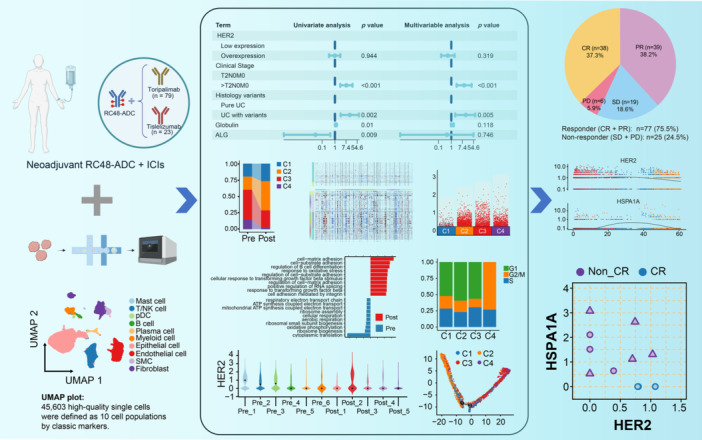

To the Editor,

Bladder cancer (BLCA) is the tenth most common cancer worldwide, with 20%–30% being muscle‐invasive bladder cancer (MIBC) [1, 2, 3]. The standard treatment for MIBC involves radical cystectomy (RC) following neoadjuvant chemotherapy based on cisplatin. Approximately 50% of the patients are ineligible for cisplatin, and due to the lack of standardized alternative regimens, their prognosis is poor [2, 4, 5]. Recent clinical trials (PURE‐01, etc.) have demonstrated promising efficacy of neoadjuvant immune checkpoint inhibitors (ICIs) in patients with MIBC [6]. Our preliminary study validates the potential of neoadjuvant chemoimmunotherapy combinations [7]. Nevertheless, effective neoadjuvant strategies remain elusive for chemotherapy‐ineligible patients and those refractory to immunotherapy.

Human epidermal growth factor receptor 2 *(HER2)* overexpression occurs in 12.4%–15% of BLCA, correlating with aggressiveness and poor prognosis [3, 8, 9]. Disitamab Vedotin (RC48‐ADC) is a novel antibody‐drug conjugate (ADC) targeting *HER2*, consisting of a recombinant humanized *HER2* IgG1 monoclonal antibody conjugated to the microtubule inhibitor monomethyl auristatin E (MMAE) via a linker [10]. It has both targeted killing and bystander effects [11, 12]. RC48‐ADC was approved in China in January 2022 for the treatment of *HER2*‐overexpression locally advanced or metastatic MIBC patients who have previously received platinum‐based chemotherapy. Multiple current clinical trials (RC48‐C005, RC48‐C009, RC48‐C011, and RC48‐C014) have shown its superior efficacy and manageable safety in advanced MIBC [13, 14, 15]. Additionally, RC48‐ADC‐based neoadjuvant treatment also holds great potential in patients with *HER2*‐positive MIBC [16].

The findings show that RC48‐ADC is a promising treatment, especially for patients resistant to immunotherapy or chemotherapy, bringing hope for better outcomes. However, few studies explore its potential in neoadjuvant treatment for MIBC. We conducted this multi‐center, real‐world study to systematically evaluate the efficacy and biomarkers of neoadjuvant RC48‐ADC combined with ICIs in patients with MIBC, providing a basis for optimizing treatment strategies.

## RESULTS AND DISCUSSION

### Efficacy of neoadjuvant RC48‐ADC combined with ICIs

**Figure 1:**
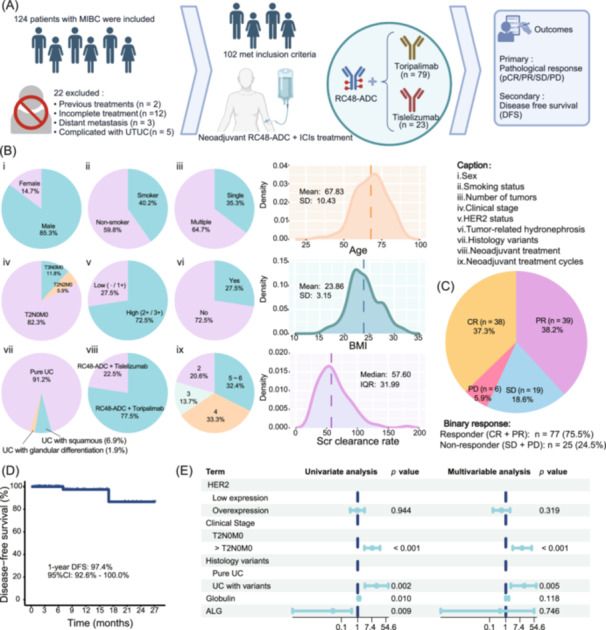
Study design, patients baseline characteristics, efficacy and relationships between clinicopathological features and pathological response. (A) Patient selection process and study design. (B) Patients baseline characteristics. (C) Pathologic response. (D) Kaplan–Meier estimates of 1‐year DFS. Two patients underwent intravesical recurrence: One patient (T2N0M0, HER2: 1+) achieved PR after receiving two cycles of neoadjuvant treatment plus maximal TURBT and had a recurrence at 7 months. The other patient (T2N0M0, HER2: 2+) reached SD after receiving six cycles of neoadjuvant treatment plus maximal TURBT and had a recurrence at 17 months. (E) Uni‐ and multivariable logistic regression of pathological response‐related factors. ALG, albumin‐to‐globulin ratio; BMI, body mass index; CR, complete response; DFS, disease‐free survival; ICIs, immune checkpoint inhibitors; IQR, inter‐quartile range; MIBC, muscle‐invasive bladder cancer; PD, progression disease; PR, partial response; Scr, serum creatinine; SD, stable disease; UC, urothelial carcinoma.

Figure 1A shows the patient selection process and study design. A total of 102 patients were finally included. Figure 1B and Table S1 present all baseline characteristics. Figure 1C and Table S2 present the distribution of patients with different pathological responses within the cohort. Thirty‐eight patients (37.3%) achieved a pathological complete response (pCR), and 39 patients (38.2%) achieved a pathological partial response (pPR). The overall rate of pathological downstaging (pCR + pPR, <pT2N0M0: responder) was 75.5% (95% confidence interval (CI): 66.0%–83.5%). A total of 6 patients (5.9%) experienced disease progression. As of January 2024, the median follow‐up time was 5.5 months (95% CI: 4.4–6.9 months). Figure 1D shows that the 1‐year disease‐free survival rate was 97.4% (95% CI: 92.6%–100.0%). Two patients underwent intravesical recurrence.

### Relationships between clinicopathological features and pathological response

The results of univariate analysis for all available clinicopathological variables between responders (CR + PR, *n* = 77) and non‐responders (SD + PD, *n* = 25) are shown in Table S3 (clinicopathological characteristics) and Table S4 (hematological characteristics). The pathological response rate in the T2N0M0 subgroup was significantly higher than that in the >T2N0M0 subgroup (83.3% vs. 38.9%, *p* < 0.001), and the response rate in pure urothelial carcinoma was higher than that in histological variants (80.6% vs. 22.2%, *p* = 0.002). Responders had a higher serum albumin‐to‐globulin ratio (*p* = 0.009) and a lower globulin level (*p* = 0.01). However, there was no significant association between *HER2* expression and pathological response (*p* = 0.944). As clearly shown in Figure 1E and Table S5, in the multivariable logistic regression analysis, there was no significant association between the hematological characteristics and the efficacy. However, the clinical stage (odds ratio (OR) = 10.08, *p* < 0.001) and histological type (OR = 13.71, *p* = 0.005) were significantly correlated with the efficacy, further confirming their important roles in predicting the efficacy.

### Single‐cell map of tumor microenvironment (TME) before and after neoadjuvant RC48‐ADC combined with ICIs

**Figure 2:**
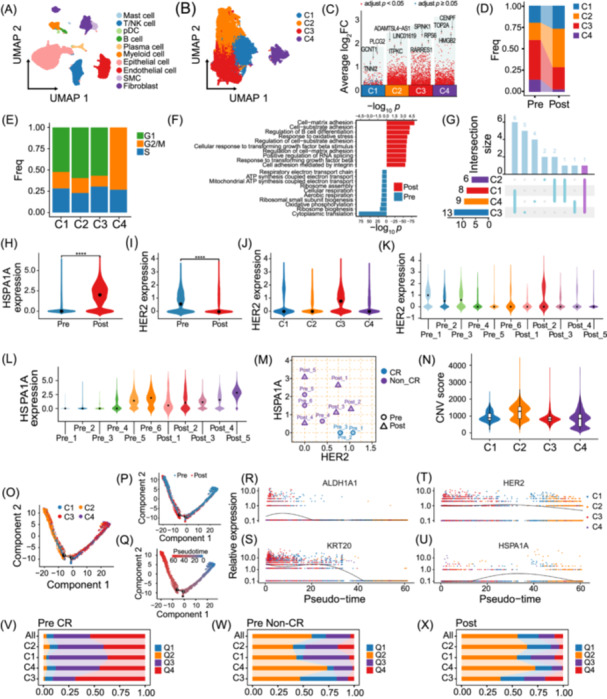
Use single‐cell RNA sequencing to explore TME dynamic changes and drug‐resistance mechanisms. (A) UMAP plot of all single cells profiled in the presenting work. Based on classic markers, 45,603 high‐quality single cells were defined as 10 cell populations: Mast cell, T/NK cell, pDC, B cell, plasma cell, myeloid cell, epithelial cell, endothelial cell, SMC, and fibroblast. (B) UMAP plot of subclusters of BLCA epithelial cells. 21,206 BLCA epithelial cells were categorized into four subclusters. Based on the differentially expressed genes among subclusters, GO and KEGG enrichment analyses were conducted. By integrating the specific functions of each subcluster, the four different subclusters were defined as follows: C1_protein refolding subcluster, C2_stress response subcluster, C3_cytoplasmic translation subcluster, and C4_cell cycle subcluster. (C) The highly expressed marker genes specific to each subcluster, shown by Manhattan plot. (D) Histogram illustrated the differences in BLCA epithelial cell population proportions before and after treatment. C3 had the highest proportion before treatment. C2 increased significantly after treatment and had the highest proportion after treatment, suggesting that C2 may resist the treatment. C4 accounted for a very small proportion after treatment, probably because it is more sensitive to treatment. (E) Histogram illustrated the differences in BLCA cell cycle phases proportions of four subclusters. The G1 phase (Gap1 phase) is the pre‐DNA synthesis stage, primarily preparing the cell for entry into the S phase and determining whether the cell meets the requirements to enter the proliferation cycle. Cells in the S phase (Synthesis phase), G2 phase (Gap2 phase), and M phase (Mitosis phase) are in an overall actively proliferating state, starting from DNA replication and ending with the completion of cell division. (F) Functional enrichment analysis highlighted the most significantly enriched pathways for all subclusters before and after treatment. Blue indicated pathways enriched before treatment, while red denoted those enriched after treatment. The enrichment analysis was based on the characteristic genes from Table S8. (G)–(L) We combined the analysis of HSPA1A and HER2 expression at different time points, in different subclusters and across various samples, along with their associations with efficacy. Results showed that only patients with high HER2 expression and no HSPA1A expression in C3 could achieve CR through treatment: (G) UpSet plot showed the intersection of overexpressed genes across four subclusters after treatment. (H) Violin plot compared the expression of HSPA1A in all BLCA epithelial cells before and after treatment. (I) Violin plot compared the expression of HER2 in all BLCA epithelial cells before and after treatment. (J) The violin plot showed the expression of HER2 in four subclusters. (K) The violin plot showed the expression of HER2 in each sample. (L) The violin plot showed the expression of HSPA1A in each sample. (M) The scatter plot showed the median values of HER2 and HSPA1A in C3 BLCA epithelial cells of each sample, and it could be seen that patients who achieved CR had the characteristics of HSPA1A‐HER2+. (N) The violin plot indicated the CNV score of four subclusters. CNV analysis has been widely used to study disease progression and development. Tumor cells generally have a higher CNV level than benign tissue cells. In tumor cells, a lower CNV is generally associated with the initial stage of tumor cell evolution, while a higher CNV is associated with a greater degree of malignancy and invasiveness. Pseudo‐time analysis of BLCA epithelial cells for all samples. BLCA epithelial cell subclusters (O), treatment groups (P) and pseudo‐temporal ordering (Q) were labeled by colors. In (Q), red represents the late stage of evolution, and blue represents the early stage of evolution. Therefore, the blue part on the right side represents the starting point of the evolution of BLCA epithelial cells, and the red part on the left side represents the end point of the evolution. All cells evolve along this trajectory from the red starting point to the blue end point. The cells at the starting point are some cells in the early stage of differentiation, while the cells at the end point are some highly differentiated cells. In (P), red represents the cells after treatment, and blue represents the cells before treatment. Most of the cells in the early stage of evolution come from the samples before treatment (on the right side), and most of the cells in the late stage of evolution come from the samples after treatment (on the left side). In (O), blue represents C1, orange represents C2, red represents C3, and purple represents C4. C3 and C4 belonged to the early stage of development (on the right side), C2 belonged to the end stage of development (on the left side), and the middle stage was dominated by C1 (in the middle). The expression of tumor stem cell markers ALDH1A1 (R) and KRT20 (S) during the evolutionary process of BLCA epithelial cells. The expression of HER2 (T) and HSPA1A (U) during the evolutionary process of BLCA epithelial cells in Pre‐CR group. Histogram illustrated the proportion in four quadrants segmented based on the expression of HER2 and HSPA1A in all epithelial cells and subclusters in Pre‐CR group (V), Pre‐NonCR group (W) and Post group (X). The division into these regions is performed as described in the legend of Figure S5. The distribution pattern of C3 in the four quadrants was generally inherited in other subclusters and was similar to the distribution of all tumor cells. TME, tumor microenvironment.

Elucidating the changes in TME before and after neoadjuvant RC48‐ADC combined with ICIs can help us understand the heterogeneity of BLCA and the differences in treatment responses. We collected 11 MIBC samples (Table S6) and performed single‐cell RNA sequencing on them. As shown in Figure 2A and Figure S1A–C, all single cells were dimensionally reduced and visualized by UMAP plots based on classic markers, clearly distinguishing 10 cell populations.

Furthermore, we categorized 21,206 BLCA epithelial cells into four clusters (Figure 2B, Figure S1D,E). Based on the differentially expressed genes among different BLCA epithelial cell subclusters (hereinafter referred to as “subclusters”) (Figure 2C, Table S7), we conducted Gene Ontology and Kyoto Encyclopedia of Genes and Genomes enrichment analyses (Figure S2A–D). By integrating the specific functions of each subcluster, we ultimately defined the four different subclusters as follows: C1_protein refolding subcluster (C1), C2_stress response subcluster (C2), C3_cytoplasmic translation subcluster (C3), and C4_cell cycle subcluster (C4). Figure 2D shows the changes in the proportion of cells of each subcluster in the TME before and after treatment.

We evaluated the cell cycle status of all tumor cells (Figure S3A). All cells in C4 were in the G2/M or S phase (Figure 2E), indicating a highly proliferative state. After treatment, the overall number of cells in the G2/M and S phases decreased (Figure S3B), but the cells in the G2/M and S phases of C2 were not inhibited (Figure S3C), suggesting the drug‐resistant characteristics of C2.

The BLCA epithelial cells were divided into two groups according to the pre‐ and post‐treatment status, differential gene and enrichment analyses being carried out (Table S8). The results showed that the tumor cells after treatment had an enhanced response to stress (Figure 2F). Figure S2E–H and Table S9 showed the further analysis of each subcluster.

### *HER2* and *HSPA1A* can be used in combination as efficacy prediction biomarkers

By analyzing the gene expression profiles after treatment, we found that *HSPA1A* was significantly overexpressed in all subclusters after treatment (Figure 2G,H and Figure S3D–F), especially in C2 (Figure S3G), suggesting a close association with treatment resistance. *HSPA1A*, a molecular chaperone protein, is expressed in various cancers, including urothelial carcinoma. It can promote the survival of tumor cells mainly through protein quality control functions and is associated with poor prognosis [17, 18].

As the target of RC48‐ADC, *HER2* showed a significantly heterogeneous distribution: it was overall highly expressed in BLCA epithelial cells before treatment (Figure 2I and Figure S3H) and specifically enriched in C3 (Figure 2J and Figure S3I). Notably, in the samples achieving CR (Pre_1‐3), the expression of *HER2* in C3 was significantly higher (median value > 0) than in other samples not achieving CR (Pre_4‐6, Post_1‐5), while *HSPA1A* was completely absent (Figure 2K–L). In addition, the high expression of *HER2* was strictly limited to C3 in all samples (Figure S4A). Although the high expression of *HSPA1A* spanned multiple subclusters, it was always accompanied by its expression in C3 (Figure S4B). The expression of *HER2* and *HSPA1A* in C3 differed among groups with different treatment efficacies (Figure S4C,D).

Based on the results, RC48‐ADC targets *HER2*‐positive tumor cells in C3, directly killed by MMAE. *HER2*‐negative tumor cells are cleared via bystander killing and immune response, achieving CR. Tumor cells with high expression of *HSPA1A* may be able to resist the microtubule inhibitory effect of MMAE, stabilize cells, and hamper bystander killing and immunity.

The clinical relevance of *HER2* and *HSPA1A* expression patterns is as follows: Only when C3 has *HER2*+ and *HSPA1A‐* (Q4, *HER2*+*HSPA1A*−), can patients achieve CR (Pre_1‐3) (Figure S5A–C). For non‐CR patients (Pre_4‐6, Post_1‐3), C3 mainly distributes in Q1 (*HSPA1A*+*HER2*+) and Q2 (*HSPA1A*+*HER2*−) (Figure S5D–K). Figure 2M shows that C3 in all samples achieved CR has *HSPA1A‐HER2+* features. In C3, patients with *HSPA1A* + (irrespective of *HER2*) don't achieve CR. This reveals the *HER2/HSPA1A* antagonistic pattern in C3 as a key efficacy predictor and biomarker. Specifically, *HER2*+*HSPA1A*− patients may benefit more from treatment. *HER2*+*HSPA1A*+ patients might need to combine other *HSPA1A*‐targeting therapies [19]. *HER2*− patients may not be suitable for RC48‐ADC and require personalized alternatives.

Previous reports indicated some *HER2*‐low/negative patients benefited from RC48‐ADC [14, 20]. First, we found an inconsistency between *HER2* test from pathological immunohistochemistry (Table S6) and single‐cell RNA sequencing (Figure 2K). More precise methods like single‐cell RNA sequencing are needed. Moreover, relying solely on *HER2* for efficacy prediction may be inaccurate. Future research should focus more on *HSPA1A* to improve the accuracy of efficacy predictions.

In our cohort, *HER2* is relatively specifically expressed in tumor cells (Figure S6A). Therefore, in centers with limited technical capabilities, it is advisable to consider using the detection results of the overall tumor tissue to substitute for the *HER2* expression. However, *HSPA1A* is not specifically expressed in tumor cells (Figure S6B). The detection results of the overall tumor tissue cannot replace the *HSPA1A* expression in tumor cells. The detection needs to be targeted at tumor cells, particularly cells in C3.

### C3 plays a dominant role in the emergence of drug‐resistance characteristics during the evolution of BLCA epithelial cells

Copy number variation (CNV) analysis demonstrated that all BLCA epithelial cells had high CNV levels (Figure S7A), confirming that the previously analyzed epithelial cells were indeed BLCA cells. C2 exhibited the highest CNV (Figure 2N), suggesting that it was at the evolutionary endpoint. Pseudo‐time analysis revealed that the four subclusters were distributed along a single evolutionary pathway: C3/C4 →C1→C2 (Figure 2O–Q and Figure S7B,C). Tumor stem cell markers ALDH1A1 and KRT20 were highly expressed in C3 and C4 during the early stage of evolution (Figure 2R–S), indicating that they possess stem cell characteristics and are the starting points of tumor evolution. Given that C4 was in the G2M/S phase, it was speculated to be the functional state of C3. Moreover, tumor cells showed remarkable characteristics of dynamic functional module transitions at different stages of evolution, suggesting that tumor cells gradually adapted to external pressures and managed to survive (Figure S7D, Table S10).

Furthermore, we found that in the Pre_CR (Figure 2T,U), Pre_NonCR (Figure S7E,F), and Post (Figure S7G,H) groups, the expression of *HER2* and *HSPA1A* remained relatively stable during the evolution of BLCA epithelial cells. The Pre_CR group maintained the characteristics of high *HER2* expression and low *HSPA1A* expression, while other groups stably exhibited the characteristics of high *HSPA1A* expression and low *HER2* expression. Figure 2V–X and Figure S8 showed the expression pattern of C3 could be inherited by other subclusters, indicating that the expression characteristics of *HER2* and *HSPA1A* originated from C3 rather than new mutations that occurred during the evolutionary process. In summary, by dominating the expression patterns of *HER2* and *HSPA1A* in BLCA, C3 determines the sensitivity and resistance to RC48‐ADC treatment.

The limitations of this study include inherent biases associated with retrospective research. Furthermore, given the recent approval of RC48‐ADC, the overall follow‐up period for this study is insufficient. This may not be adequate to fully reflect the long‐term efficacy, recurrence risk, and late adverse events. Longer‐term follow‐up will be carried out in the future to verify the durability and safety of the treatment. As RC48‐ADC is a newly approved drug in the market and its applicable population is not extensive enough, only 102 patients were included in our study. However, among the studies with reported data, our sample size is relatively large. Finally, RC48‐ADC is mainly used in China at present, and it may still take some time to be promoted globally.

## CONCLUSION

Neoadjuvant RC48‐ADC combined with ICIs showed promising efficacy in patients with MIBC. Predicting treatment efficacy based on single‐cell RNA sequencing is feasible. This study will expand neoadjuvant treatment strategies for patients who are ineligible for neoadjuvant chemotherapy.

## AUTHOR CONTRIBUTIONS

**Jiao Hu**: Conceptualization; methodology; resources; writing—original draft; writing—review and editing. **Luzhe Yan**: Investigation; resources; visualization; writing—original draft; writing—review and editing. **Jinhui Liu**: Software; data curation; formal analysis; writing—original draft; writing—review and editing. **Minfeng Chen**: Conceptualization; methodology; resources; writing—original draft; writing—review and editing. **Peihua Liu**: Investigation; resources. **Dingshan Deng**: Software; validation. **Chaobin Zhang**: Investigation; data curation. **Yunbo He**: Validation; writing—review and editing. **Benyi Fan**: Methodology; resources. **Huihuang Li**: Software; visualization. **Guanghui Gong**: Visualization; software. **Jiatong Xiao**: Investigation; validation. **Ruizhe Wang**: Data curation; formal analysis. **Xiao Guan**: Resources; investigation. **Shiyu Tong**: Validation; visualization. **Yangle Li**: Methodology; software. **Nannan Li**: Resources; investigation. **Zhiwang Tang**: Formal analysis; validation. **Teng Zhang**: Software; visualization. **Hao Li**: Software; visualization. **Bin Huang**: Visualization; software. **Ning Gao**: Software; visualization. **Wei He**: Methodology; validation. **Zhiyong Cai**: Methodology; validation. **Yifan Liu**: Investigation; data curation. **Zefu Liu**: Formal analysis; visualization. **Yu Gan**: Validation; software. **Yu Cui**: Resources; writing—review and editing. **Yuanqing Dai**: Methodology; validation. **Yi Cai**: Methodology; validation. **Zhenyu Nie**: Methodology; validation. **Zhenyu Ou**: Conceptualization; project administration; supervision; writing—review and editing. **Jinbo Chen**: Conceptualization; project administration; supervision; writing—review and editing. **Xiongbing Zu**: Conceptualization; supervision; funding acquisition; writing—review and editing.

## CONFLICT OF INTEREST STATEMENT

The authors declare no conflicts of interest.

## ETHICS STATEMENT

This study was approved by the Ethics Committees of all centers. The authors are accountable for all aspects of the work in ensuring that questions related to the accuracy or integrity of any part of the work are appropriately investigated and resolved. Written informed consents were obtained from participants or their immediate families.

## Supporting information


**Figure S1.** Perform dimensionality reduction, clustering, and definition on all cells and BLCA epithelial cells of all samples.
**Figure S2.** Functional enrichment analysis of BLCA epithelial cells.
**Figure S3.** Cell cycle analysis and the expression of HSPA1A and HER2 in BLCA epithelial cells.
**Figure S4.** The expression of HER2 and HSPA1A in four BLCA epithelial cell subclusters in each sample and different groups.
**Figure S5.** The expression of HER2 and HSPA1A in all C3 BLCA epithelial cells of all samples
**Figure S6.** The expression of HER2 and HSPA1A in all single cells.
**Figure S7.** CNV analysis and the expression of HER2 and HSPA1A during the evolutionary process of BLCA epithelial cells.
**Figure S8.** Histogram illustrated the proportion in four quadrants segmented based on the expression of HER2 and HSPA1A in all BLCA epithelial cells and each subcluster in each sample.

**Table S1.** Patients baseline characteristics.
**Table S2.** Pathologic response.
**Table S3.** Univariate analysis of clinical characteristics and pathological response.
**Table S4.** Univariate analysis of hematological features and pathological response.
**Table S5.** Uni‐ and multivariable logistic regression of pathological response‐related factors.
**Table S6.** Baseline characteristics of patient samples used for single ‐ cell RNA sequencing.
**Table S7.** Differentially expressed genes of four BLCA subclusters.
**Table S8.** Differentially expressed genes of all BLCA cells before and after treatment.
**Table S9.** Differentially expressed genes of four BLCA subclusters before and after treatment.
**Table S10**. GO biological process enrichment analysis of gene modules based on pseudo‐temporal expression patterns.

## Data Availability

The data that support the findings of this study are available from the corresponding author upon reasonable request. The data are available from the author upon request. All relevant data supporting the key findings of this study are available within the article and its supplementary information files. The codes during the current study are available at https://github.com/jnhv/2025scRNA. Other data are available from the corresponding author upon reasonable request. Due to ethical and legal restrictions, individual‐level data from single‐cell RNA sequencing cannot be made publicly available. Data are available upon request to the corresponding author and subject to local rules and regulations. Supplementary materials (methods, figures, tables, graphical abstract, slides, videos, Chinese translated version, and update materials) may be found in the online DOI or iMeta Science http://www.imeta.science/.
