# Supplementary material for: Efficacy and biomarker analysis of neoadjuvant disitamab vedotin (RC48‐ADC) combined immunotherapy in patients with muscle‐invasive bladder cancer: A multi‐center real‐world study
Source: Imeta. 2025 Apr 14;4(3):e70033. doi: 10.1002/imt2.70033 (PMC12130573; doi:10.1002/imt2.70033)
Supplement: Supplementary file 1 — Figure S1. Perform dimensionality reduction, clustering, and definition on all cells and BLCA epithelial cells of all samples. Figure S2. Functional enrichment analysis of BLCA epithelial cells. Figure S3. Cell cycle analysis and the expression of HSPA1A and HER2 in BLCA epithelial cells. Figure S4. The expression of HER2 and HSPA1A in four BLCA epithelial cell subclusters in each sample and different groups. Figure S5. The expression of HER2 and HSPA1A in all C3 BLCA epithelial cells of all samples Figure S6. The expression of HER2 and HSPA1A in all single cells. Figure S7. CNV analysis and the expression of HER2 and HSPA1A during the evolutionary process of BLCA epithelial cells. Figure S8. Histogram illustrated the proportion in four quadrants segmented based on the expression of HER2 and HSPA1A in all BLCA epithelial cells and each subcluster in each sample. [file IMT2-4-e70033-s002.docx]

**Supporting information to:**

**Efficacy and biomarker analysis of neoadjuvant disitamab vedotin (RC48-ADC) combined immunotherapy in patients with muscle-invasive bladder cancer: A multi-center real-world study**

**Running title:** Neoadjuvant RC48-ADC combined immunotherapy for muscle-invasive bladder cancer

Jiao Hu^1,2^^,7#^, Luzhe Yan^1,2,7#^, Jinhui Liu^1,2,7#^, Minfeng Chen^1,2,7#^, Peihua Liu^1,2,7^, Dingshan Deng^1,2,7^, Chaobin Zhang^1,2,7^, Yunbo He^1,2,7^, Benyi Fan^1,2,7^, Huihuang Li^1,2,7^, Guanghui Gong^3^, Jiatong Xiao^1,2,7^, Ruizhe Wang^1,2,7^, Xiao Guan^1,2,7^, Shiyu Tong^1,2,7^, Yangle Li^1,2,^^7^, Nannan Li^4^, Zhiwang Tang^4^, Teng Zhang^5^, Hao Li^5^, Bin Huang^6^, Ning Gao^6^, Wei He^1,2,7^, Zhiyong Cai^1,2,7^, Yifan Liu^1,2,7^, Zefu Liu^1,2,7^, Yu Gan^1,2,7^, Yu Cui^1,2,7^, Yuanqing Dai^1,2,7^, Yi Cai^1,2,7^, Zhenyu Nie^1,2,7^, Zhenyu Ou^1,2,7,*^, Jinbo Chen^1,2,^^7,*^, Xiongbing Zu^1,2,7,8,*^

^1^Department of Urology, Xiangya Hospital, Central South University, Changsha, 410008, China

^2^National Clinical Research Center for Geriatric Disorders, Xiangya Hospital, Central South University, Changsha, 410008, China

^3^Department of Pathology, Xiangya Hospital, Central South University, Changsha, 410008, China

^4^Department of Urology, The First Hospital of Changsha, Changsha, 410005, China

^5^Department of Urology, First People’s Hospital of Guiyang, Guiyang, 550002, China

^6^Department of Urology, Xiangya Boai Rehabilitation Hospital, Changsha, 410100, China

^7^Furong Laboratory, Changsha, 410008, China

^8^Department of Urology, Hunan Provincial People's Hospital, the First Affiliated Hospital of Hunan Normal University, Changsha, 410005, China.

^#^These authors contributed equally: Jiao Hu, Luzhe Yan, Jinhui Liu, and Minfeng Chen

***Correspondence:** zuxbxy@csu.edu.cn (Xiongbing Zu), ouzhenyu1@163.com (Zhenyu Ou) & chenjinbo@csu.edu.cn (Jinbo Chen)

**Materials and Methods**

**Study design, outcomes and participants selection**

A multicenter real-world retrospective study was conducted to assess the efficacy of RC48-ADC, a recombinant humanized anti-HER2-MMAE conjugate developed by RemeGen, Ltd., combined with ICIs as neoadjuvant therapy in MIBC. This study was reviewed and approved by our Institutional Ethics Committee. Written informed consents were obtained from participants or their immediate families. All data were collected by two trained researchers. The primary outcome was pathological downstaging, including complete pathological response (pCR: pT0N0M0) and partial response (pPR: pTa, Tis, and T1N0M0). pT2-4N0M0 with an enlarged tumor volume, pN+, and pM+ were defined as progressive disease (PD), while the remaining status was defined as stable disease (SD). The secondary outcome was the 1-year disease free survival (DFS). The inclusion Criteria were as follows: 1) Patients with pathologically confirmed MIBC. 2) Patients refusing or intolerant of cisplatin-based chemotherapy and chemoimmunotherapy. 3) Received neoadjuvant RC48-ADC combined with ICIs and had not received any other systemic treatment. 4) HER2 expression 0~3+. 5) Patients receiving RC or maximal transurethral resection of the bladder tumor after neoadjuvant treatment for pathologic staging. 6) Regular follow-up during treatment. Exclusion Criteria: 1) Previous neoadjuvant chemotherapy or immunotherapy. 2) Did not receive a complete neoadjuvant treatment. 3) Distant metastasis. 4) Concurrent upper tract urothelial carcinoma. 5) Lost to follow-up.

**Patient Treatment Protocol**

All patients underwent imaging examinations based on computed tomography (CT) or magnetic resonance imaging (MRI) scan before treatment to assess the clinical stage. All patients included into this study were administered RC48-ADC at a dose of 2.0 mg/kg, with a maximum dose of 120 mg, via intravenous infusion over 60-90 minutes every 2 weeks. Concurrently, tislelizumab was administered intravenously at 200 mg every three weeks or toripalimab at 3 mg/kg every two weeks. After two cycles of treatment, a CT/MRI scan was performed to assess the primary treatment response. If there is an obvious tumor remission from CT/MRI scans, continue with up to 6 cycles of neoadjuvant treatment. For patients whose tumor response was not obvious (SD) as evaluated by CT and those with disease progression (PD), the neoadjuvant treatment was discontinued and radical cystectomy (RC) was performed. If patients presented with intolerable adverse reactions, the treatment was immediately terminated. Since these patients refused to receive cisplatin-based chemotherapy or were not suitable for it, we recommended that these patients undergo RC. Patients with a significant response to neoadjuvant therapy will be assessed and the choice of bladder-preserving treatment will be based on their wishes. Follow-up evaluations were conducted at the third month post-surgery, including blood biochemistry tests, CT/MRI scans, and other relevant assessments. Subsequently, the patients were monitored every 3–6 months for 2 years. Patients who remained disease-free within the first 2 years postoperatively were followed up annually.

**HER2 testing**

HER2 testing was completed by professional doctors from the Department of Pathology, Xiangya Hospital, Central South University.This study utilized the *VENTANA anti-HER2/neu (4B5) Rabbit Monoclonal Primary Antibody* to detect the expression of HER2. All the samples were properly pre-processed according to the standard procedures. Immunohistochemical staining was performed on tissue samples in accordance with the instructions of this kit. The interpretation criteria for HER-2 protein expression referred to the 2018 ASCO/CAP Breast Cancer Testing Guidelines, with slight modifications based on the staining characteristics of urothelial carcinoma: Negative (no staining & less than 10% of tumor cell membranes with incomplete and weak staining); 1+ (≥10% of tumor cell membranes with incomplete and weak staining); 2+ (≥10% of tumor cell membranes with weak to moderate-intensity complete membrane staining & less than 10% of tumor cell membranes with complete strong staining); 3+ (≥10% of tumor cell membranes with complete strong staining). The HER2 expression of each sample was jointly evaluated by two professional pathologists. In this study, HER2 IHC 2+ and 3+ were defined as HER2 overexpression, and HER2 IHC negative and 1+ were defined as low expression.

The main limitations include: 1) The technique has relatively high requirements for the operation process and the professional quality of the personnel; 2) Improper tissue processing (such as errors in the fixation, freezing and other steps) may generate artificial artifacts, antibody trapping or false negative results; 3) Excessive or insufficient counterstaining may affect the correct interpretation of the results; 4) Non-immunological binding of proteins or reaction products of the substrate may lead to false positive results; 5) In immunohistochemical tests, a negative result indicates that the antigen has not been detected, rather than the absence of the antigen in the measured cells or tissues.

**Biomarker analysis based on single-cell RNA sequencing**

**Collection and processing of biological samples**

We collected bladder tumor specimens from patients before and after neoadjuvant RC48-ADC combined with ICIs. The eleven samples in our study were from ten patients, with one patient contributing samples both pre- and post- treatment. Six samples were collected before neoadjuvant treatment (three patients achieved CR after treatment: Pre_1, Pre_2, Pre_3; the other three patients did not achieve CR after treatment: Pre_4, Pre_5, Pre_6), and five samples were collected after treatment (Post_1-Post_5). The detailed procedures for single-cell isolation, sequencing, and raw data processing have been outlined in previous articles.[1] Following quality control checks with an RNA integrity number (RIN ≥ 7.0) and cell viability (≥ 85%), we successfully obtained six bladder tumor specimens via transurethral resection prior to treatment and five specimens from transurethral resection of bladder tumor (TURBT) or RC post-treatment for analysis.

**Preprocessing, dimensionality reduction, clustering, and annotation of single-cell transcriptomic data**

FASTQ files were aligned to the GRCh38 human reference genome utilizing Cell Ranger software (version 7.0.1) from 10x Genomics, with unique molecular identifier (UMI) counts tabulated for each barcode. Analysis of the UMI count matrix was performed using the Seurat R package[2] (version 4.3.0). Cells exhibiting fewer than 1000 UMIs or more than 20% mitochondrial UMIs were deemed low-quality and excluded. Potential doublets were identified using the DoubletFinder R package[3] (version 2.0.3), with an anticipated doublet rate set at 0.8% of 1,000 cells. After quality control and the removal of doublet cells, a total of 45,603 cells were used for subsequent analysis.

Gene expression data normalization involved the NormalizeData function, applying the "LogNormalize" global-scaling normalization method, which adjusts each cell’s gene expression measurements based on total expression, scales up by a factor of 10,000, and log-transforms the results. The top 2000 highly variable genes (HVGs) were determined using Seurat’s FindVariableGenes function. The PCA analysis was performed using the "RunPCA" function with the default parameters.

To address the batch effects in single-cell RNA-sequencing data, the Harmony algorithm was implemented, which was carried out based on the "Integration_SCP" function of the SCP R package. Clustering was conducted using the "FindNeighbors" and "FindClusters" functions with 40 principal components (PCs) at a resolution of 0.6, and each cluster was annotated according to the known markers. These 45,603 single cells were classified into 10 cell populations: Mast cell ("KIT", "CPA3", "TPSAB1"), T/NK cell ("CD3D", "CD3E", "CD3G"), pDC ("CLEC4C", "IL3RA", "LILRA4"), B cell ("MS4A1", "CD19", "CD79A"), Plasma cell ("MZB1", "IGHG1", "JCHAIN"), Myeloid cell ("LYZ", "CD14", "CD68"), Epithelial cell ("EPCAM", "KRT18", "KRT19"), Endothelial cell ("PECAM1", "VWF", "CD34"), SMC ("TAGLN", "MYH11", "CNN1"), and Fibroblast ("COL1A1", "COL1A2", "COL3A1"). Cell visualization was achieved through the UMAP algorithm via the RunUMAP function, using the same PCs as those used for clustering.

**Subclustering of BLCA epithelial cells and annotation**

Utilizing established epithelial cell markers ("EPCAM", "KRT18", "KRT19") , we identified a total of 21,206 cells which were then subjected to re-clustering. Following the same preprocessing workflow described earlier, then we used the "FindNeighbors" and "FindClusters" functions on 15 principal components (PCs) with a resolution of 0.1 to perform the clustering.

The marker genes of each epithelial subcluster were identified using the "FindAllMarkers" function of the Seurat package, with the parameter min.pct = 0.25. Genes with adjusted *p*-values < 0.05 and log_2_FC > 0.5 were identified as significantly differentially expressed marker genes.

Following re-clustering, Gene Ontology (GO) and Kyoto Encyclopedia of Genes and Genomes (KEGG) pathway analyses were performed for each subcluster based on Top 100 DEGs in each cluster. By focusing on genes that were highly expressed within each subcluster, we systematically assigned names to the subclusters based on their functional characteristics.

Specifically, the following lists the main functions enriched based on GO and KEGG analyses for four subclusters.

C1_protein refolding subcluster: protein refolding, chaperone cofactor−dependent protein refolding, 'de novo' post−translational protein folding, 'de novo' protein folding, protein processing in endoplasmic reticulum, etc.

C2_stress response subcluster: p53 signaling pathway, integrated stress response signaling, apoptosis, intrinsic apoptotic signaling pathway, mitophagy − animal, etc.

C3_cytoplasmic translation subcluster: cytoplasmic translation, ribosome, ribosome biogenesis, ribosome assembly, rRNA processing, etc.

C4_cell cycle subcluster: mitotic nuclear division, chromosome segregation, sister chromatid segregation, nuclear division, regulation of chromosome segregation, etc.

**Differentially expressed genes analysis of various grouping methods of BLCA epithelial cells.**

To precisely examine the gene expression differences in tumor cells, we first divided all tumor cells into two groups based on the pre - and post - treatment status and obtained the DEGs. Then, within each tumor cell subtype, we also divided the cells into two groups according to the pre - and post - treatment status for DEG analysis. The DEG analysis between two cellular populations was conducted using the 'FindAllMarkers' function with the parameter min.pct = 0.25. DEGs were defined with the thresholds of avg_log_2_FC > 1 and *p*_val_adj < 0.05. All the DEGs were used for subsequent functional enrichment analysis.

To explore the commonalities among the post - treatment grouped tumor cells, we took the intersection of the positive DEGs in the post - treatment group of each tumor cell subtype to identify the shared genes. To focus on a smaller number of more important genes with greater differences, we set the thresholds as avg_log_2_FC > 2.5 and *p*_val_adj < 0.05.

**Gene Ontology (GO) and Kyoto Encyclopedia of Genes and Genomes (KEGG) enrichment analysis**

GO and KEGG analyses were conducted on the DEGs using the ‘ClusterProfiler’ (4.2.2) R package.[4] For each analysis, gene sets were evaluated for over-representation among cluster markers or DEGs by calculating enrichment *p*-values using the 'enricher' function (default parameters) based on the hypergeometric distribution. Hypergeometric *p*-values were adjusted for multiple testing in each instance using the Benjamini-Hochberg correction.

**inferCNV analysis**

Initial CNVs for each region were estimated using the inferCNV (1.10.1) R package (https://bioconductor.org/packages/infercnv) with default parameters. Epithelial cells were selected as malignant cells, while T/NK cells and myeloid cells were used as the normal reference cells. De-noising processes were then applied to generate the final CNV profiles.

**Cell cycle analysis**

The prediction of the cell cycle phase for individual cells was conducted using the CellCycleScoring function in Seurat R package. Genetic markers specific to the G2/M and S phases were utilized to assign scores to cells. Cells that did not express markers for either the G2/M or S phases were categorized as being in the G1 phase.

**Pseudotime analysis**

Developmental pseudotime was determined using the Monocle2 (2.22.0) R package.[5] The differentialGeneTest function within Monocle2 was utilized to identify ordering genes (q-value < 0.01), which were likely informative for arranging cells along the pseudotime trajectory. Dimensional reduction and clustering analysis were conducted using the reduceDimension function, followed by trajectory inference via the orderCells function with default settings. Gene expression changes across pseudotime were visualized using the plot_genes_in_pseudotime function.

Gene expression dynamics during tumor cell evolution were analyzed. Genes detected in ≥10 cells were retained to ensure robust expression quantification. Pseudotime-associated genes were systematically identified by applying the "differentialGeneTest" function in Monocle R package. A false discovery rate (FDR)-adjusted threshold (q-value < 0.01) was applied to define genes with statistically significant temporal dynamics. The top 500 ranked genes were partitioned into four co-expression modules via k-means clustering and visualized in a heatmap using row-scaled z-score-normalized expression values by using the function "plot_pseudotime_heatmap".

Perform Gene Ontology Biological Process (GO BP) enrichment analysis on the genes of each cluster. Sort the results in ascending order of the "adj *p* value" in the positive direction. Select the top 5 entries among those with ‘adj *p* value < 0.05’ for visualization.

**Statistical analysis and visualization**

The normality of the quantitative data was assessed using the Shapiro–Wilk test. Data that followed a normal distribution were described using mean ± standard deviation, whereas non-normally distributed data were summarized using median and interquartile range. Categorical variables are described using counts and proportions. Categorical variables were compared between the two groups using the chi-square test or Fisher's exact test, as appropriate. For continuous variables, the *t*-test was used for normally distributed data and the Wilcoxon test was applied for non-normal data. All data visualization and statistical analyses were conducted using R software (version 4.1.2). Statistical significance was defined as a two-sided *p*-value of < 0.05. The ggplot2 and ggpubr R packages (https://rpkgs.datanovia.com/ggpubr/) were used for basic plotting, while the UpSet R package[6] was employed to create upset plots and Venn diagrams. The SCP R package (https://github.com/zhanghao-njmu/SCP) was utilized for partial single-cell data analysis and visualization.

**Supplementary figures:**

**Figure S1.** Perform dimensionality reduction, clustering, and definition on all cells and BLCA epithelial cells of all samples.

(A)UMAP plot of all single cells showing clusters by samples.

(B)Dotplot showed the expression of marker genes in the cell types. The color of the dots represented the expression levels of the markers, while dot size indicates fraction of expressing cells.

(C)Histogram indicated the proportion of all cell types in tumor tissue of each sample.

(D)UMAP plot of BLCA epithelial cells showing subclusters by samples.

(E)Histogram indicated the proportion of BLCA epithelial cell subclusters in tumor tissue of each sample.

**Figure S2.** Functional enrichment analysis of BLCA epithelial cells.

(A)-(D)Functional enrichment analysis demonstrated the most significantly enriched functions for each subclusters. Blue represented KEGG pathways, while red represented GO pathways. The enrichment analysis was performed based on the top 100 marker genes for each subcluster. The enrichment analysis was based on the characteristic genes from Table S7.

(E)-(H)Functional enrichment analysis highlighted the most significantly enriched pathways for each subclusters before and after treatment. Blue indicated pathways enriched before treatment, while red denoted those enriched after treatment.

The enrichment analysis was based on the characteristic genes from Table S9.

**Figure S3.** Cell cycle analysis and the expression of HSPA1A and HER2 in BLCA epithelial cells.

(A)UMAP plot of cell cycle of BLCA epithelial cells.

(B)Histogram indicated the difference of cell cycle phases of BLCA epithelial cells before and after treatment.

(C)Histogram showed the proportion distribution of cell cycle phases in four subclusters before and after treatment.

(D)Venn diagram showed the intersection of overexpressed genes across four subclusters after treatment.

(E)UMAP plot of HSPA1A expression of BLCA epithelial cells before and after treatment.

(F)Violin plot showed the expression of HSPA1A in four subclusters before and after treatment.

(G)Violin plot showed the expression of HSPA1A in four subclusters.

(H)UMAP plot of HER2 expression of BLCA epithelial cells before and after treatment.

(I)Violin plot showed the expression of HER2 in four subclusters before and after treatment.

**Figure S4.** The expression of HER2 and HSPA1A in four BLCA epithelial cell subclusters in each sample and different groups.

(A)Violin plot showed the expression of HER2 in four subclusters in each sample. If a subcluster contained fewer than 5 cells, that subcluster was excluded from the analysis.

(B)Violin plot showed the expression of HSPA1A in four subclusters in each sample. If a subcluster contained fewer than 5 cells, that subcluster was excluded from the analysis.

(C)Box plot showed the differences in the expression of HER2 in C3 among different efficacy groups.

(D)Box plot showed the differences in the expression of HSPA1A in C3 among different efficacy groups.

**Figure S5.** The expression of HER2 and HSPA1A in all C3 BLCA epithelial cells of all samples. We introduced the "jitter" technique to reduce the overlap between data points. By comparing the expression levels of HER2 and HSPA1A with 0, four quadrant regions were defined: Q1 represents HER2>0 and HSPA1A>0; Q2 represents HER2≤0 and HSPA1A>0; Q3 represents HER2≤0 and HSPA1A≤0; and Q4 represents HER2>0 and HSPA1A≤0. Notably, when drawing the dashed lines that delineate different regions, to accurately reflect the regions to which each data point belongs, the positions of these dashed lines were adjusted accordingly to match the scatter points that have undergone jitter processing. In each subplot, green dots represent individual cells, while the red diamond points indicate the median values of HER2 and HSPA1A expression across all cells. The values in the four corner regions represent the proportion of cells in each region relative to the total number of cells.

(A)-(C)The expression of HER2 and HSPA1A in all C3 BLCA epithelial cells of the three samples in the Pre_CR group.

(D)-(F)The expression of HER2 and HSPA1A in all C3 BLCA epithelial cells of the three samples in the Pre_NonCR group.

(G)-(K)The expression of HER2 and HSPA1A in all C3 BLCA epithelial cells of the five samples in the Post group.

**Figure S6.** The expression of HER2 and HSPA1A in all single cells.

(A)UMAP plot of the expression of HER2 in all cells.

(B)UMAP plot of the expression of HSPA1A in all cells.


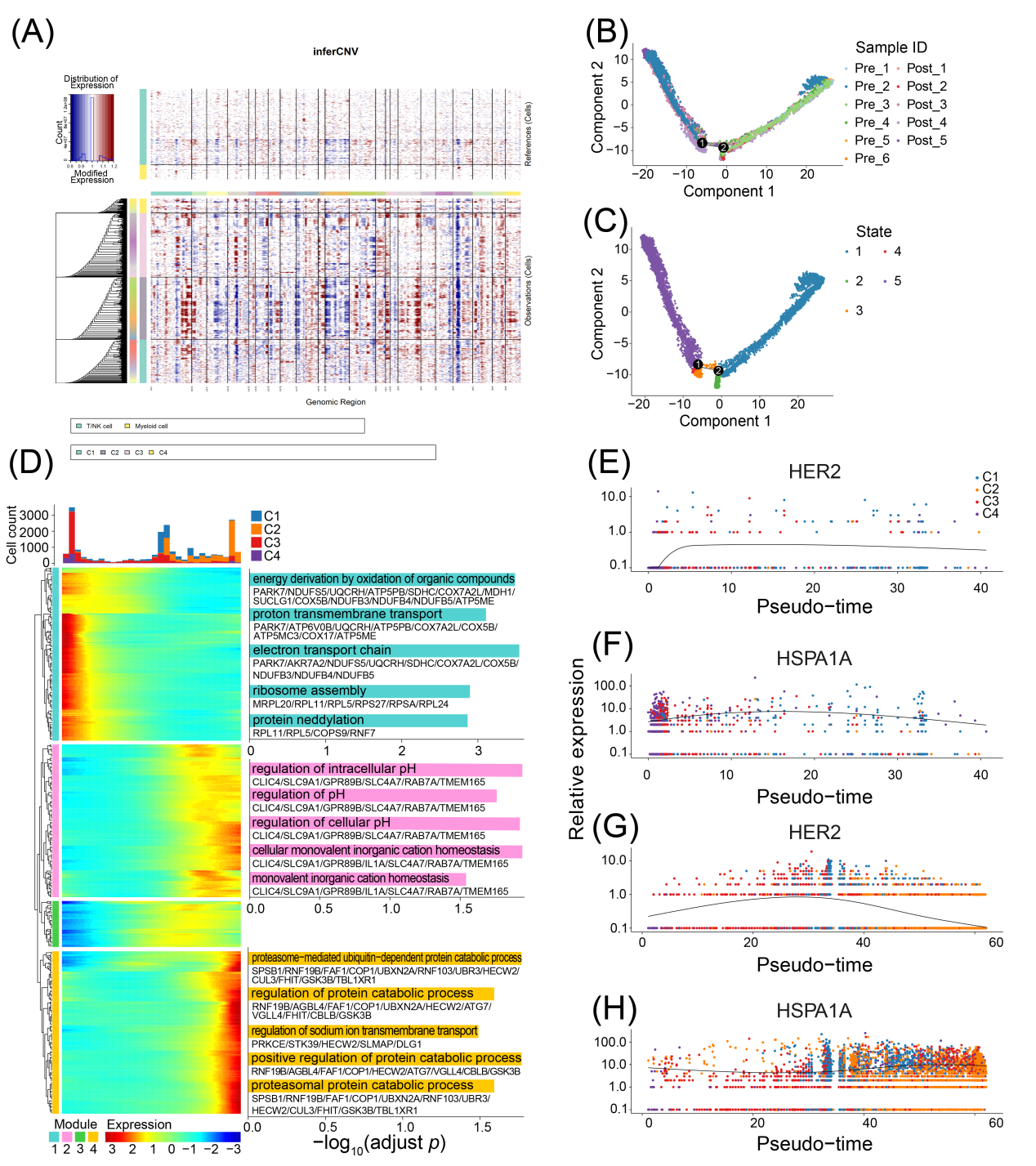


**Figure S7.** CNV analysis and the expression of HER2 and HSPA1A during the evolutionary process of BLCA epithelial cells.

(A)Heatmap represented the CNV analysis for all BLCA epithelial cells.

(B)-(C)Pseudo-time analysis of BLCA epithelial cells for all samples. Samples ID and state were labeled by colors.

(D)Heatmap showed that BLCA epithelial cells exhibit remarkable dynamic functional module transformation characteristics at different stages of evolution. Select the top 5 entries among those with an adjusted *p*-value < 0.05 from the complete results in Table S10 for visualization. Module 1 mainly includes the electron transport chain, energy derivation by oxidation of organic compounds, proton transmembrane transport, ribosome assembly, and protein neddylation, which are related to energy metabolism and protein synthesis. In the early stage, tumor cells may proliferate actively and require a large amount of energy and protein synthesis. Module 2 is mainly involved in cellular monovalent inorganic cation homeostasis, regulation of intracellular pH, and regulation of cellular pH. In the middle and late stages of evolution, the TME may become acidified, and tumor cells need to regulate the pH and ion balance to adapt to the environment and promote survival and invasion. Module 4 mainly contains the proteasome-mediated ubiquitin-dependent protein catabolic process, positive regulation of the protein catabolic process, proteasomal protein catabolic process, and regulation of sodium ion transmembrane transport. In the terminal stage, it involves more regulation of protein degradation and cellular homeostasis, which may be related to how tumor cells respond to stress and resist RC48-ADC treatment. This evolutionary pattern indicates that in the early stage, BLCA epithelial cells rely on metabolic reprogramming to drive malignant proliferation. In the late stage, they adapt to the pressure on the TME from the outside and maintain a survival advantage by dynamically coordinating the mechanisms of ion homeostasis and protein turnover. The activation of the protein degradation pathway specific to the terminal stage may provide potential targets for treating drug resistance.

(E)-(F)The expression of HER2 (E) and HSPA1A (F) during the evolutionary process of BLCA epithelial cells in Pre-NonCR group.

(G)-(H)The expression of HER2 (G) and HSPA1A (H) during the evolutionary process of BLCA epithelial cells in Post group.

**Figure S8.** Histogram illustrated the proportion in four quadrants segmented based on the expression of HER2 and HSPA1A in all BLCA epithelial cells and each subcluster in each sample. The division into these regions is performed as described in the legend of Figure S5. If a subcluster contains fewer than 5 cells, that subcluster was excluded from the analysis.

**References：**

1. Chen, Zhaohui, Lijie Zhou, Lilong Liu, Yaxin Hou, Ming Xiong, Yu Yang, Junyi Hu, and Ke Chen. 2020. "Single-cell RNA sequencing highlights the role of inflammatory cancer-associated fibroblasts in bladder urothelial carcinoma." *Nature Communication* 11:5077. <https://doi.org/10.1038/s41467-020-18916-5>.

2. Hao, Yuhan, Stephanie Hao, Erica Andersen-Nissen, William M Mauck 3rd, Shiwei Zheng, Andrew Butler, Maddie J Lee, et al. 2021. "Integrated analysis of multimodal single-cell data." *Cell* 184:3573-3587 e3529. <https://doi.org/10.1016/j.cell.2021.04.048>.

3. McGinnis, Christopher S, Lyndsay M Murrow, and Zev J Gartner. 2019. "DoubletFinder: Doublet Detection in Single-Cell RNA Sequencing Data Using Artificial Nearest Neighbors." *Cell Systems* 8:329-337 e324. <https://doi.org/10.1016/j.cels.2019.03.003>.

4. Yu, Guangchuang, Li-Gen Wang, Yanyan Han, and Qing-Yu He. 2012. "clusterProfiler: an R Package for Comparing Biological Themes Among Gene Clusters." *OMICS: A Journal of Integrative Biology* 16:284-287. <https://doi.org/10.1089/omi.2011.0118>.

5. Qiu, Xiaojie, Qi Mao, Ying Tang, Li Wang, Raghav Chawla, Hannah A Pliner, and Cole Trapnell. 2017. "Reversed graph embedding resolves complex single-cell trajectories." *Nature Methods* 14:979-982. <https://doi.org/10.1038/nmeth.4402>.

6. Conway, Jake R, Alexander Lex, and Nils Gehlenborg. 2017. "UpSetR: an R package for the visualization of intersecting sets and their properties." *Bioinformatics* 33:2938-2940. <https://doi.org/10.1093/bioinformatics/btx364>.
